# Neurotrophin-4 promotes the specification of trophectoderm lineage after parthenogenetic activation and enhances porcine early embryonic development

**DOI:** 10.3389/fcell.2023.1194596

**Published:** 2023-07-13

**Authors:** Mirae Kim, Joohyeong Lee, Lian Cai, Hyerin Choi, Dongjin Oh, Ali Jawad, Sang-Hwan Hyun

**Affiliations:** ^1^ Veterinary Medical Center and College of Veterinary Medicine, Laboratory of Veterinary Embryology and Biotechnology (VETEMBIO), Chungbuk National University, Cheongju, Republic of Korea; ^2^ Institute of Stem Cell and Regenerative Medicine (ISCRM), Chungbuk National University, Cheongju, Republic of Korea; ^3^ Graduate School of Veterinary Biosecurity and Protection, Chungbuk National University, Cheongju, Republic of Korea

**Keywords:** neurotrophin-4, blastocyst, embryonic development, parthenogenesis, pig

## Abstract

Neurotrophin-4 (NT-4), a neurotrophic factor, appears to affect early embryonic development because it is secreted not only by neurons but also by oviductal and uterine epithelial cells. However, no studies have characterized the effects of NT-4 on early embryonic development in pigs. In this study, we applied the experimental model of parthenogenetic-activation (PA)-derived embryos. Herein, we investigated the effect of NT-4 supplementation during the *in vitro* culture (IVC) of embryos, analyzed the transcription levels of specific genes, and outlined the first cell lineage specification for porcine PA-derived blastocysts. We confirmed that NT-4 and its receptor proteins were localized in both the inner cell mass (ICM) and trophectoderm (TE) in porcine blastocysts. Across different concentrations (0, 1, 10, and 100 ng/mL) of NT-4 supplementation, the optimal concentration of NT-4 to improve the developmental competence of porcine parthenotes was 10 ng/mL. NT-4 supplementation during porcine IVC significantly (*p* < 0.05) increased the proportion of TE cells by inducing the transcription of TE lineage markers (*CDX2*, *PPAG3*, and *GATA3* transcripts). NT-4 also reduced blastocyst apoptosis by regulating the transcription of apoptosis-related genes (*BAX* and *BCL2L1* transcripts) and improved blastocyst quality via the interaction of neurotrophin-, Hippo-yes-associated protein (Hippo-YAP) and mitogen-activated protein kinase/extracellular regulated kinase (MAPK/ERK) pathway. Additionally, NT-4 supplementation during IVC significantly (*p* < 0.05) increased *YAP1* transcript levels and significantly (*p* < 0.01) decreased *LATS2* transcript levels, respectively, in the porcine PA-derived blastocysts. We also confirmed through fluorescence intensity that the YAP1 protein was significantly (*p* < 0.001) increased in the NT-4-treated blastocysts compared with that in the control. NT-4 also promoted differentiation into the TE lineage rather than into the ICM lineage during porcine early embryonic development. In conclusion, 10 ng/mL NT-4 supplementation enhanced blastocyst quality by regulating the apoptosis- and TE lineage specification-related genes and interacting with neurotrophin-, Hippo-YAP-, and MAPK/ERK signaling pathway during porcine *in vitro* embryo development.

## 1 Introduction

*In vitro* embryo production (IVP) can have applications ranging from the treatment of human reproductive disorders to gamete preservation in animals of high genetic conservation value, thereby helping to understand early embryonic development in mammals (Rizos et al., 2008). Pigs have become transgenic model animals and potential organ donors for xenotransplantation because of their physiological similarities to humans (Abeydeera, 2002). The production of successful transgenic pigs depends on acquiring high-quality mature oocytes and zygotes using IVP techniques. Over the past few decades, there have been efforts to increase the efficiency of IVP of pig embryos. Despite this, the efficiency of IVP is significantly lower than that of *in vivo* production, with approximately 30%–40% of the fertilized porcine oocytes developing into the blastocyst stage (Chen et al., 2021). Inadequate *in vitro* culture (IVC) conditions manifest as a lack of various growth factors, high oxygen content (>21% O_2_), high temperature (>42 °C), and inadequate osmolality (<270 or >300 mOsm), acting as stressors for porcine *in vitro* embryonic development (Chen et al., 2021). The *in vivo* pre-implantation embryos are exposed to various growth factors expressed in the follicles, oviducts, and endometrium (Richter, 2008). Various growth factors (e.g., insulin-like growth factor-I, epidermal growth factor, fibroblast growth factor, and granulocyte-macrophage colony-stimulating factor) and cytokines play paracrine and/or autocrine roles during early embryonic development and implantation (Brison and Schultz, 1998; Hardy and Spanos, 2002; Dey et al., 2004). In early embryonic development, growth factors can act in an autocrine manner to control embryonic growth and differentiation, or the embryos can produce growth factors that act as paracrine factors by regulating endometrial receptivity for implantation (Kawamura et al., 2007). As pigs have the protracted implantation period (days 14–19), it is a suitable animal model for studying autocrine and/or paracrine interactions between the conceptus (embryo and extra-embryonic membranes) and endometrium during the implantation period (Ziecik et al., 2011).

Mammalian embryos undergo mitosis and eventually develop into blastocysts with two distinct cell lineages: the inner cell mass (ICM) and trophectoderm (TE). The ICM becomes a fetus and the TE develops into placental trophoblasts. In particular, the TE differentiates into extravillous cytotrophoblasts and syncytiotrophoblasts, mediating implantation in the uterine wall during embryonic development (Schulz et al., 2008). The trophoblast, which is the first epithelial layer of the blastocysts, forms the placenta; this layer is in direct contact with the uterine fluid, and it transports nutrients and energy substrates to the ICM (Pfeffer and Pearton, 2012). In addition, these cells also play an important role in the implantation and formation of the maternal–fetal interface (Red-Horse et al., 2004). Although the trophoblast plays an essential role in initiating implantation and successful pregnancy, the underlying molecular mechanisms responsible for TE lineage specification in pigs remain unclear.

Neurotrophins regulate the survival, growth, proliferation, differentiation, death, and development of neuronal and non-neuronal cells (Bibel and Barde, 2000; Kim et al., 2004). There are four neurotrophin families: nerve growth factor (NGF), brain-derived neurotrophic factor (BDNF), neurotrophin-3 (NT-3), and neurotrophin-4 (NT-4). Neurotrophins are associated with implantation, maintenance of pregnancy, regulation of placental angiogenesis, and fetal brain development (Dhobale, 2014; Sahay et al., 2017; D’Angelo et al., 2020). NT-4 is a neurotrophic factor related to the transforming growth factor β superfamily (Seifer et al., 2002). It is an intra-ovarian growth factor derived from granulosa cells in the mammalian ovary and promotes oocyte maturation in pigs (Kim et al., 2021; Kim et al., 2022). Among the families of neurotrophins, both BDNF and NT-4 bind to tropomyosin receptor tyrosine kinase B (TrkB), a high-affinity receptor, and p75 neurotrophin receptor (p75^NTR^), a low-affinity receptor, and these are known to play physiologically similar roles in cortical development (McAllister et al., 1995) and oocyte maturation (Seifer et al., 2002). BDNF and NT-4 mRNA transcripts and proteins are expressed in mouse trophoblast cells (Kawamura et al., 2007) and the placenta (Kawamura et al., 2009). Addition of BDNF (40 ng/mL) during bovine IVC significantly (*p* < 0.05) increased blastocyst formation rates after *in vitro* fertilization (IVF) and parthenogenetic activation (PA), and it was also demonstrated the BDNF protein is localized in the TE cells of bovine blastocysts but not in the ICM (Yi et al., 2008). Lim et al. demonstrated that BDNF enhances the proliferation of porcine endometrial epithelial cells by suppressing endoplasmic reticulum (ER) stress during early pregnancy through the activation of PI3K and MAPK signaling pathways (Lim et al., 2017). However, no study has investigated the effects of NT-4, which has physiological effects similar to that of BDNF, on pre-implantation embryonic development, implantation, and maintenance of pregnancy in pigs.

Our previous studies have demonstrated the effects of NT-4 on IVM of oocytes in the porcine reproductive system (Kim et al., 2021; Kim et al., 2022). As NT-4 plays an important role in porcine follicular development and oocyte maturation, we further evaluated and confirmed whether NT-4 improved IVP efficiency by affecting early embryonic development. Since NT-4 is expressed in mouse TE, placental, oviductal, and uterine epithelial cells (Kawamura et al., 2007; Kawamura et al., 2009), it has been shown that it may affect embryonic development. Therefore, we hypothesized that the supplementation of NT-4 during IVC will have a positive effect on early embryonic development in pigs. In this study, we confirmed whether NT-4 and its receptors (i.e., Total-TrkB, Phospho-TrkB, and p75^NTR^) are expressed in porcine PA-derived blastocysts via differential immunostaining. We investigated the effects of NT-4 on embryonic development after PA and examined the relative mRNA transcription levels of specific genes in NT-4-treated blastocysts. The Hippo and Notch pathways control the first-cell fate decision process in mammalian embryos (Rayon et al., 2014). Specifically, the Hippo-YAP signaling pathway is one of the key regulators for dividing the ICM/TE lineage in mammalian embryos (Nishioka et al., 2008; Hirate et al., 2013; Lorthongpanich and Issaragrisil, 2015). It has been demonstrated that during the first cell lineage specification of mouse embryos, the Hippo and Notch pathways converge on *Cdx2* to regulate TE lineage specification (Rayon et al., 2014). In particular, YAP1, a downstream effector of the Hippo pathway, can participate in the regulation of cell proliferation and differentiation during embryonic development while regulating transcription of genes such as *CTGF* (Zhao et al., 2008). Both *YAP1* and *LATS2* (a negative regulator of the *YAP1*) transcripts are essential for the expression of genes involved in porcine early embryonic development and the segregation of the ICM and TE lineages (Emura et al., 2020). Therefore, we evaluated the expression of specific cell lineage markers (SOX2 and CDX2) and Hippo effector (YAP1) in PA-derived blastocysts through immunostaining.

## 2 Materials and methods

### 2.1 Chemicals and reagents

Recombinant human NT-4 (450-04) was purchased from PeproTech (Rocky Hill, NJ, United States). NT-4 protein was used after being dissolved in Dulbecco’s phosphate-buffered saline (DPBS; LB001-02; WELGENE, Gyeongsan, Gyeongsangbuk-do, Republic of Korea) with 0.1% (w/v) bovine serum albumin, as previously described (Kim et al., 2022). Verteporfin (HY-B0146; MedChemExpress, Princeton, NJ, United States), a yes-associated protein (YAP) inhibitor, was then dissolved in dimethyl sulfoxide (DMSO; Sigma Aldrich Corporation, St. Louis, MO, United States). All chemicals and reagents used in this study were purchased from Sigma–Aldrich Corporation, unless otherwise indicated.

### 2.2 Oocyte collection and IVM

Pig ovaries were collected from a local slaughterhouse and transported to the laboratory within 3 h in 0.9% (w/v) NaCl at 37°C–39°C. Oocyte collection and IVM were performed as previously described (Kim et al., 2021). The ovaries were washed twice with 0.9% (w/v) NaCl, and cumulus-oocytes complexes (COCs) were aspirated from 3–6-mm follicles using a 10-mL disposable syringe with an 18-G needle attached. The solution was allowed to settle at 37 C for 5°min, supernatant was removed, and precipitate was resuspended in HEPES-buffered Tyrode’s medium containing 0.05% (w/v) polyvinyl alcohol (TLH-PVA). After washing the precipitate of follicular fluids twice with TLH-PVA, the COCs were observed using a stereomicroscope (SZX-ILLK100; Olympus Optical Co., Ltd., Tokyo, Japan), and those with a homogeneous cytoplasm and more than three layers of compact cumulus cells were selected. Approximately 50–60 randomly selected COCs were transferred to each well of a 4-well dish (Nunc, Roskilde, Denmark) containing 500 μL of IVM medium (TCM199; Gibco, Grand Island, NY, United States) supplemented with 0.6 mM cysteine, 0.91 mM sodium pyruvate, 10 ng/mL epidermal growth factor, 75 μg/mL kanamycin, 1 μg/mL insulin, and 10% (v/v) porcine follicular fluid. For the first 22 h of IVM, the COCs were incubated in maturation medium containing 10°IU/mL equine chorionic gonadotropin and 10°IU/mL human chorionic gonadotropin, followed by incubation without equine and human chorionic gonadotropins for the next 20 h. All IVM procedures were performed in a humidified incubator (Astec, Fukuoka, Japan) at 39 C with 5% CO_2_.

### 2.3 PA and IVC of embryos

After IVM, the cumulus cells of COCs were denuded using 0.1% hyaluronidase. For the PA experiment, the metaphase II (MII) oocytes (oocytes with first polar body extrusion) were specifically selected and washed with calcium-free TLH-PVA and used. For activation, the MII oocytes were washed in 280 mM mannitol solution containing 0.01 mM CaCl_2_ and 0.05 mM MgCl_2_. Then, the MII oocytes were placed between electrodes in a chamber covered with the activation medium (260 mM mannitol solution containing 0.001 mM CaCl_2_ and 0.05 mM MgCl_2_) and activated with two direct-current pulses of 120 V/mm for 60 μs using an electrical pulsing machine (LF101; Nepa Gene, Chiba, Japan). The electrically activated oocytes were incubated for 4 h in the IVC medium (porcine zygote medium; PZM-3) containing 5 μg/mL cytochalasin B at 39°C in a humidified atmosphere of 5% CO_2_ and 95% N_2_. Subsequently, the PA embryos were washed in a fresh IVM medium, transferred into droplets of 30 μL of the fresh IVC medium (10 gametes/drop), and covered with mineral oil. During the IVC procedures, the IVC medium was supplemented with NT-4 at concentrations of 0, 1, 10, and 100 ng/mL for each group (Kim et al., 2021). The “0 ng/mL group” is the control group in which only DPBS containing 0.1% bovine serum albumin (BSA) as a vehicle was added without NT-4. For the YAP inhibition experiment, we first dissolved DMSO (0.1%) and DPBS (2%) in the PZM-3 medium; these mixtures were then used as vehicles. Porcine zygotes cultured in the IVC medium were supplemented with NT-4, verteporfin, and NT-4 + verteporfin for 7 days. PA embryos were cultured at 39 °C for 7 days under a humidified atmosphere of 5% O_2_, 5% CO_2_, and 90% N_2_. The IVC medium was replaced with 30 μL of fresh PZM-3 droplets after 48 h (day 2).

### 2.4 Evaluation of embryo quality and total cell counts

To evaluate developmental potency, the cleavage of embryos was evaluated on day 2 after PA. The normally cleaved embryos were categorized into three groups with two to three cells, four to five cells, and six to eight cells. On day 7, the blastocysts were categorized into three groups according to the degree of expansion and hatching status as early, expanded, and hatched, as previously described (Yoon et al., 2019a). To examine the blastocyst quality, the total cell number of blastocysts was counted after staining with 10 μg/mL Hoechst-33342 for 5 min. The stained blastocysts of each group were mounted on glass slides in a drop of 100% glycerol and examined using an epifluorescence microscope (TE300; Nikon, Tokyo, Japan).

### 2.5 Quantitative reverse transcription-polymerase chain reaction (qRT-PCR)

At least 20 blastocysts from each group (control and NT-4-treated group) were collected in 1.5 mL microcentrifuge tubes (SPL Life Sciences, Co. Ltd., Pocheon, Gyeonggi-do, Republic of Korea), washed with DPBS and stored at −80 °C until analysis. Total RNA was extracted from blastocysts using the TRIzol reagent (TaKaRa Bio, Inc., Otsu, Shiga, Japan). cDNA synthesis was performed using a 5× reverse transcription master mixture (Elpis Bio, Inc., Chungcheongnam-do, Daejeon, Republic of Korea) following the manufacturer’s protocol. To perform qRT-PCR, the synthesized cDNA (0.5 μg/μL) was mixed with 2× SYBR Premix Ex Taq (TaKaRa Bio Inc.) and 10 pmol of specific primers (Macrogen, Inc., Seoul, Republic of Korea). The primers used in this experiment are listed in Supplementary Table S1. The qRT-PCR analysis was carried out using a CFX96 Touch real-time PCR detection system (Bio-Rad, Hercules, CA, United States), and the reactions were performed as follows: pre-denaturation at 95 °C for 5 min and 40 cycles of denaturation at 95 °C for 15°s, annealing at 57 °C for 15°s, and extension at 72 °C for 30 s. Data were collected at the extension phase of each cycle, and the relative quantification (R) value was calculated using the following equation: R = 2^-[ΔCtsample−ΔCtcontrol]^. Normalization of gene expression levels was performed using the 18S ribosomal RNA (*RN18S*) gene as the control.

### 2.6 Immunofluorescence staining—Localization of YAP1, NT-4 and its receptors; ICM/TE cell counts

After PA, blastocysts were stained with Hoechst-33342 to count total cell numbers and differentially immunostained with SOX2 (ICM marker) and CDX2 (TE marker) antibodies to determine the ICM/TE ratio. PA-derived blastocysts were washed using DPBS with CaCl_2_ and MgCl_2_ (LB001-01; WELGENE) containing 0.1% (w/v) PVA and fixed with 4% paraformaldehyde for 30 min at room temperature (RT). The fixed blastocysts were washed with DPBS containing 0.1% PVA for 5 min and permeabilized with the permeabilization solution (DPBS containing 0.1% PVA supplemented with 0.5% Triton X-100) for 1 h at RT. After fixation and permeabilization, the blastocysts were washed twice with DPBS containing 0.1% PVA and treated with Image-iT™ FX signal enhancer (I36933; Invitrogen, Carlsbad, CA, United States) for 30 min to reduce non-specific background signals and increase target signal intensity. Then, the blastocysts were incubated with the blocking buffer (12411S; Cell Signaling Technology, Danvers, MA, United States) for 1 h at RT and co-incubated with appropriately diluted primary antibodies in the blocking buffer overnight at 4°C. The antibodies used in this experiment are listed in Supplementary Table S2. On the following day, the blastocysts were washed three times with DPBS containing 0.1% PVA using a shaker (120 rpm) for 5 min each. Subsequently, appropriately diluted secondary antibodies in blocking buffer were added to the blastocysts and incubated at RT for 2 h using a shaker. The stained blastocysts were washed thrice with DPBS containing 0.1% PVA, then counterstained with 10 μg/mL Hoechst-33342 for 5 min, and mounted on glass slides in a drop of anti-fade solution (Molecular Probes, Inc., Eugene, OR, United States). Each Hoechst-33342-positive cell (Total cell number of blastocysts) was classified as ICM or TE based on the predominant expression of SOX2 (Green) or CDX2 (Red), respectively. The stained blastocysts were examined via confocal microscopy (Carl Zeiss, Thornwood, NY, United States), and all images were analyzed using the ZEN (blue edition, Carl Zeiss) software program. Briefly, to measure fluorescence intensity, a circular region of interest was drawn along the circumference of each blastocyst and the average intensity of that region was calculated using the ZEN software (Yin et al., 2021). The mean fluorescence intensity was then normalized via Hoechst-33342 fluorescence (White et al., 2016).

### 2.7 Statistical analysis

All experiments were carried out at least three times. Statistical analyses were conducted using GraphPad Prism version 7.0 (GraphPad Software, Inc., San Diego, CA, United States). A one-way analysis of variance, followed by Duncan’s multiple range test, was performed to examine the percentage of embryonic development data (e.g., the rate of cleavage and blastocyst formation and total cell number of blastocysts). An unpaired two-tailed Student’s *t*-test was conducted to evaluate data of the remaining experiments, excluding that of the percentage of embryonic development. All data are reported as mean ± standard error of the mean. Statistical significance was set at *p* < 0.05.

## 3 Results

### 3.1 Identification and localization of NT-4 and its receptors in porcine PA-derived blastocysts

**FIGURE 1:**
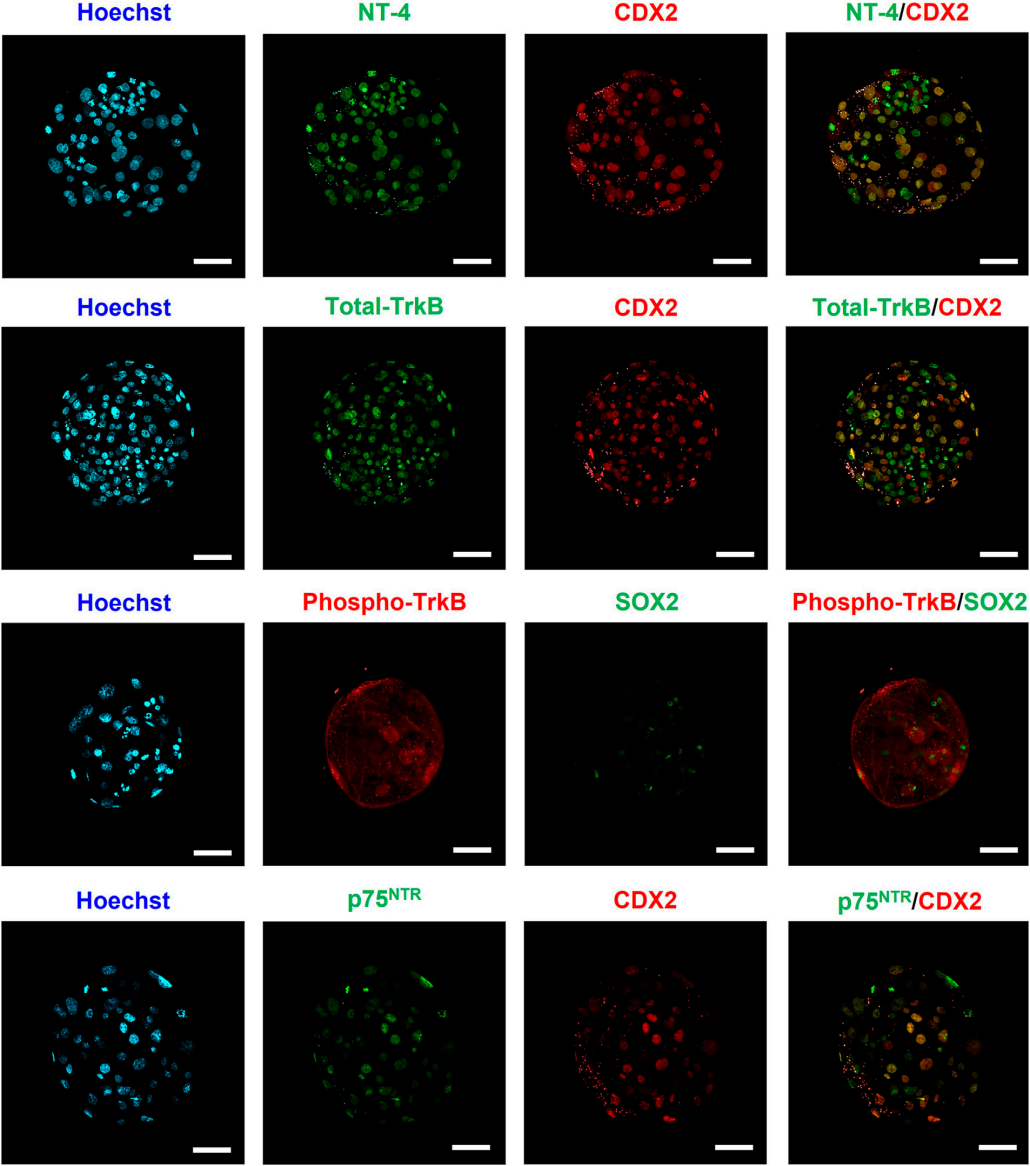
Identification and localization of NT-4 and its receptors in porcine parthenogenetic activation (PA)-derived blastocysts via immunofluorescence analysis. Immunofluorescence staining images of SOX2, CDX2, NT-4, Total-TrkB, Phospho-TrkB, and p75^NTR^. In blastocysts, SOX2-expressing cells are candidates for the inner cell mass, whereas CDX2-expressing cells are candidates for the trophectoderm. Alexa Flour (AF) 488 fluorescence is shown in green and AF594 fluorescence is shown in red. Nuclei were counterstained with Hoechst-33342 (blue). Scale bars = 50 μm.

Immunofluorescence staining was performed to identify the localization of NT-4 and its receptors (Total TrkB, Phospho-TrkB, and p75^NTR^) in porcine PA-derived blastocysts (Figure 1). We performed differential immunostaining using either SOX2 or CDX2 antibodies to distinguish between ICM and TE within the blastocysts. In the blastocysts, SOX2-expressing cells are candidates for the ICM, whereas CDX2-expressing cells are candidates for the TE. NT-4 and its receptors were confirmed to be present in both the ICM and the TE cells in porcine PA-derived blastocysts. Phospho-TrkB was distributed throughout the surface and cytoplasm of the blastocysts, whereas NT-4, total-TrkB and p75^NTR^ were localized to the nuclei of the blastocysts.

### 3.2 Developmental competence of PA embryos supplemented with various concentrations of NT-4 during porcine IVC

**FIGURE 2:**
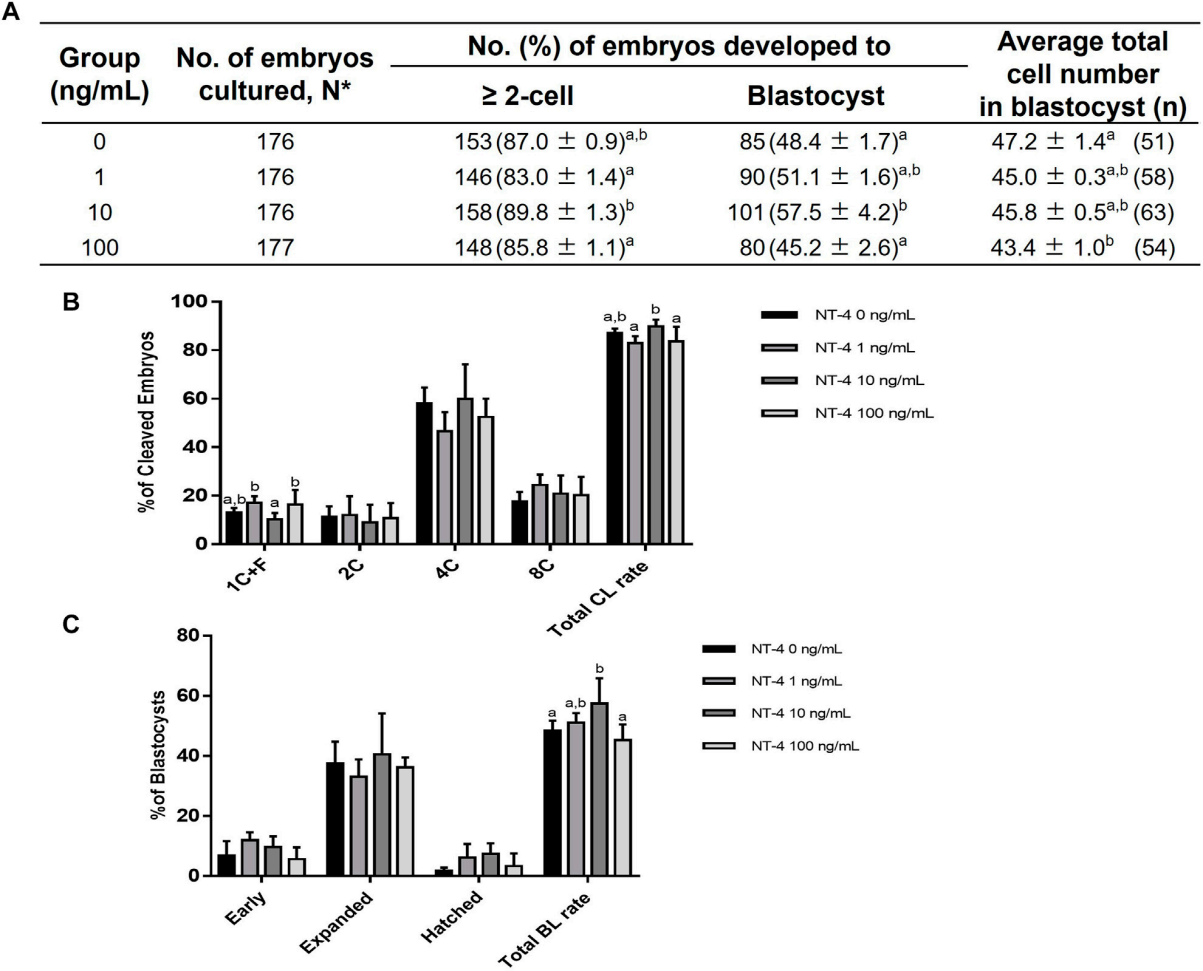
Effect of various concentrations of NT-4 supplementation during *in vitro* culture (IVC) on embryonic development after parthenogenetic activation (PA). **(A)** Effect of NT-4 supplementation during IVC on embryonic development after PA. **(B)** Effect of NT-4 supplementation during IVC on the cleavage pattern of PA embryos at day 2. The cleavage rate is the percentage of cleaved embryos on day 2 of IVC. **(C)** Effect of NT-4 supplementation during IVC on the percentage of PA embryos that developed to the blastocyst stage at day 7. The blastocyst formation rate refers to the percentage of embryos that developed to the blastocyst stage. The average total number of blastocysts is the average number of nuclei per blastocyst. Within each end point, bars with different letters **(A,B)** are significantly different (*p* < 0.05) for different concentrations of NT-4 treatment. 1 + C: 1 cell + fragmentation embryos; 2C: 2 cells; 4C: 4 cells; 8C: 8 cells; CL: cleavage; BL: blastocyst. The experiment was replicated four times.

We determined the optimal NT-4 concentration for porcine IVC by analyzing the developmental competence of PA embryos after treatment with various concentrations (0, 1, 10, and 100 ng/mL) of NT-4 for 0–7 days (Figure 2A). The cleavage rate is the percentage of cleaved embryos on day 2 of IVC. On day 2 after PA, no significant differences were observed in the cleavage rate of all NT-4 treatment groups (83.0% ± 1.4%, 89.8% ± 1.3%, and 85.8% ± 1.1%, respectively) compared with that of the control (87.0% ± 0.9%) (Figures 2A,B). The blastocyst formation rate refers to the percentage of embryos that developed to the blastocyst stage. When NT-4 was treated at a concentration of 100 ng/mL (43.4 ± 1.0, n = 54) during IVC, the average total cell number of blastocysts was significantly (*p* < 0.05) lowered compared to those of control (47.2 ± 1.4, n = 51) (Figure 2A). However, supplementation with 1- (45.0 ± 0.3, n = 58) and 10 ng/mL (45.8 ± 0.5, n = 63) NT-4 groups during IVC did not affect the average total cell number of blastocysts compared to the control (Figure 2A). Blastocyst formation rates were significantly higher (*p* < 0.05) in the 10 ng/mL NT-4-supplemented group (57.5% ± 4.2%, n = 101) than in the control (48.4% ± 1.7%, n = 85) (Figures 2A,C). Therefore, after setting the optimal NT-4 concentration for IVC of porcine PA embryos as 10 ng/mL, additional experiments were performed only for the control and 10 ng/mL NT-4 treatment groups (Figure 2).

### 3.3 Effects of NT-4 supplementation during porcine IVC on the expression of specific genes in PA embryos

**FIGURE 3:**
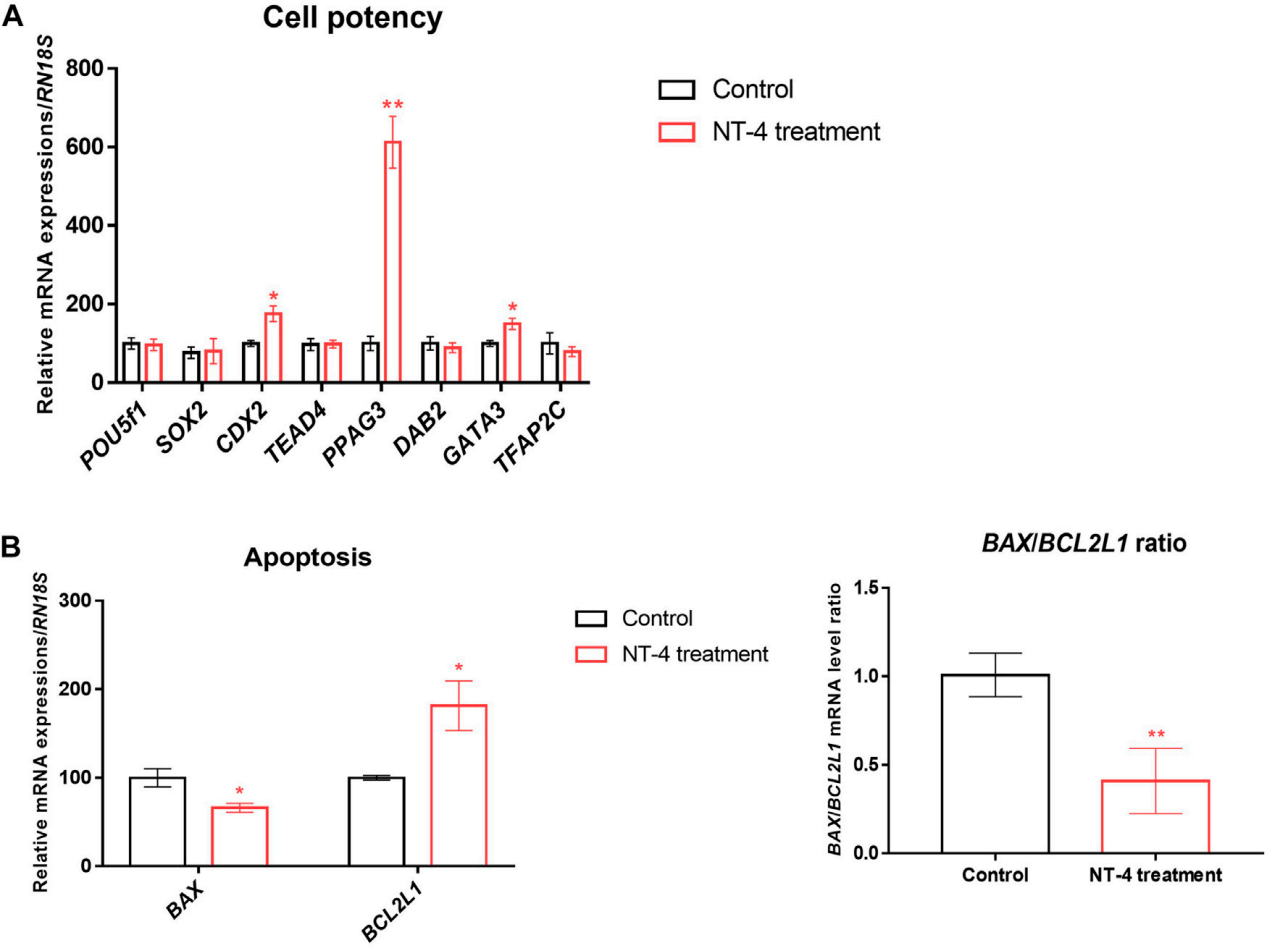
Relative mRNA expression levels of cell potency- and apoptosis-related genes in NT-4-treated blastocysts. **(A)** Mean ± SEM expression of *POU5F1*, *SOX2*, *CDX2*, *TEAD4*, *PPAG3*, *DAB2*, *GATA3*, and *TFAP2C* mRNA in PA-derived blastocysts. **(B)** Mean ± SEM expression of *BAX* and *BCL2L1* mRNA in PA-derived blastocysts. Data were analyzed by Student’s t-tests. Asterisks indicate statistical significance (*p < 0.05 and **p < 0.01). This experiment was replicated at least three times.

**FIGURE 4:**
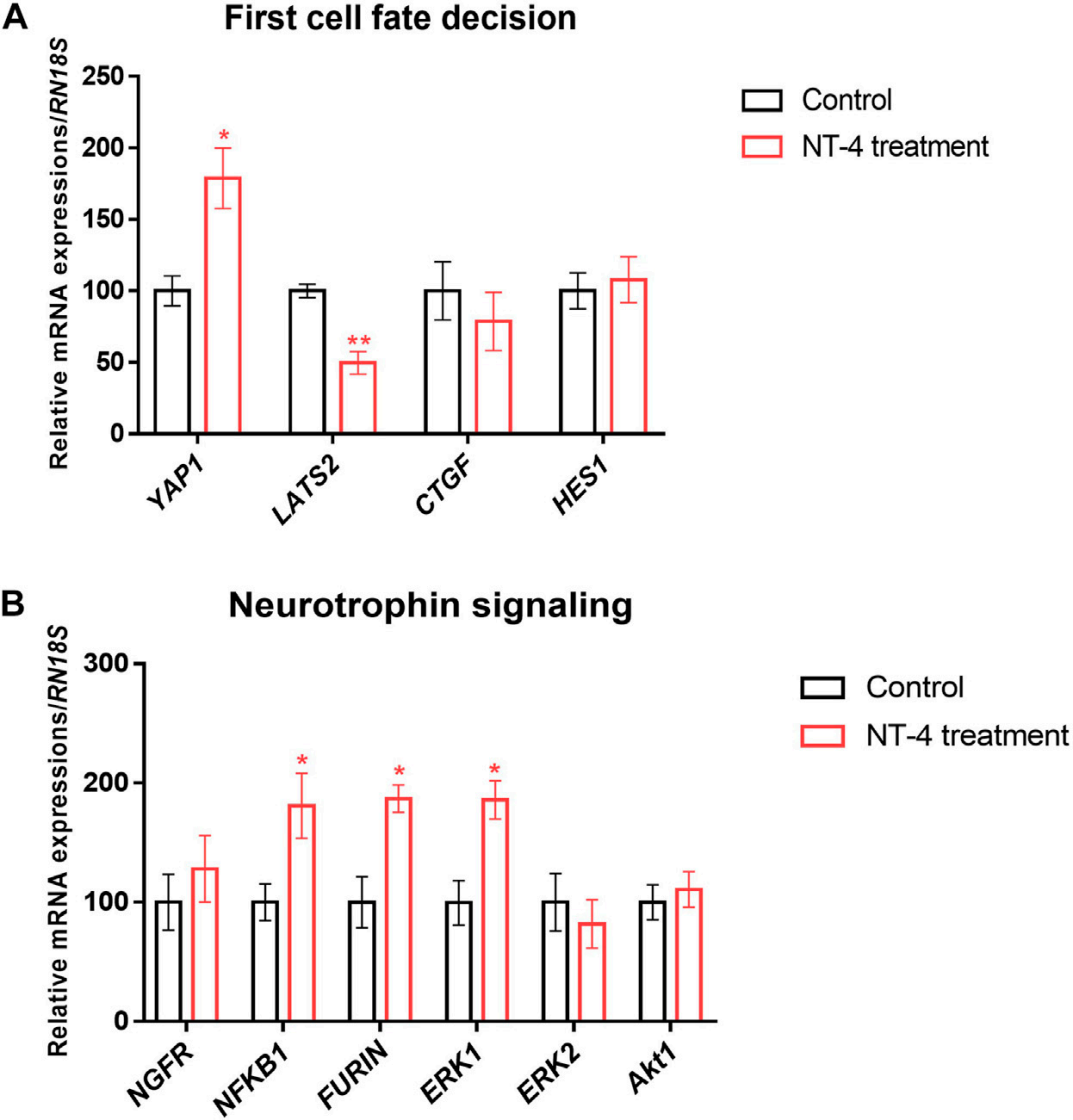
Relative mRNA expression of first-cell fate decision- and neurotrophin signaling-related genes in NT-4-treated blastocysts. **(A)** Mean ± SEM expression of *YAP1*, *LATS2*, *CTGF*, and *HES1* mRNA in PA-derived blastocysts. **(B)** Mean ± SEM expression of *NGFR*, *NFKB1*, *FURIN*, *ERK1*, *ERK2*, and *Akt1* mRNA in PA-derived blastocysts. Data were analyzed by Student’s t-tests. Asterisks indicate statistical significance (*p < 0.05 and **p < 0.01). This experiment was replicated at least three times.

To examine the effect of NT-4 on transcription patterns during IVC, the mRNA expression levels of cell potency-, apoptosis-, first-cell fate decision-, and neurotrophin signaling-related genes were evaluated in the PA-derived blastocysts of the NT-4-supplemented (10 ng/mL) and control groups. As shown in Figure 3A, no significant difference was observed in mRNA expression levels of the pluripotent-related genes (also known as ICM-related genes; *POU5F1* and *SOX2* transcripts) between the NT-4-supplemented and control groups. However, the transcript levels of *CDX2* (*p* < 0.05), *PPAG3* (*p* < 0.01), and *GATA3* (*p* < 0.05), which are known TE-related genes, were significantly increased in the NT-4-supplemented blastocysts. The mRNA expression level of the anti-apoptotic gene *BCL2L1* was significantly increased (*p* < 0.05), whereas those of the pro-apoptotic gene *BAX* (*p* < 0.05) and the *BAX*/*BCL2L1* ratio (*p* < 0.01) were significantly reduced in the NT-4-supplemented group (Figure 3B). Moreover, we investigated the mRNA expression levels of genes associated with the Hippo (*YAP1*, *CTGF*, and *LATS2* transcripts) and Notch (*HES1* transcript) pathways, which regulate the first embryonic cell decisions (Figure 4A). No significant difference was observed in the mRNA expression level of both *HES1* (an effector of the Notch pathway) and *CTGF* (YAP1 target gene) between NT-4-treated and control blastocysts. However, the transcript level of *YAP1*, a downstream effector of the Hippo pathway, was significantly increased (*p* < 0.05) in NT-4-supplemented blastocysts, whereas that of *LATS2*, a negative regulator of the *YAP1*, was significantly decreased (*p* < 0.01) in NT-4-supplemented blastocysts (Figure 4A). In addition, among the neurotrophin signaling-related genes, the mRNA expression level of *NFKB1* (a downstream gene of NGFR), *FURIN* (a proprotein convertase), and *ERK1* (a member of the mitogen-activated protein kinase; MAPK pathway) were significantly increased (*p* < 0.05) in the NT-4-supplemented group (Figure 4B).

### 3.4 Effects of NT-4 supplementation during porcine IVC on ICM and TE blastocyst cell numbers and ICM/TE ratio

**FIGURE 5:**
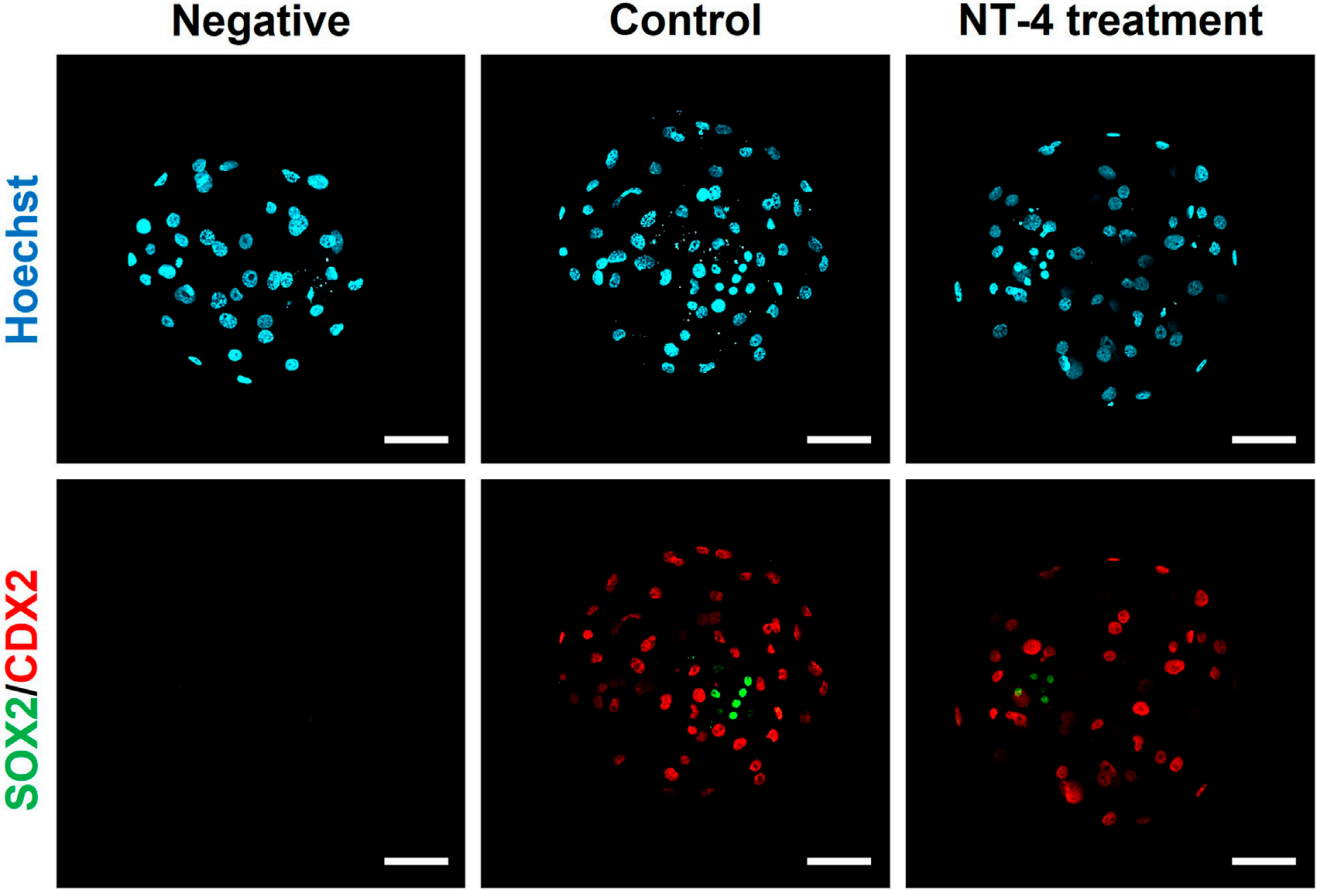
Dual immunofluorescence images of porcine parthenogenetic activation (PA)-derived blastocysts. NT-4 was added at 0 (Control) and 10 ng/mL during PA embryo development *in vitro*. Green (AF488) fluorescence labeling indicates SOX2. Red (AF594) fluorescence labeling indicates CDX2. Nuclei were counterstained with Hoechst-33342 (blue). A negative control followed all the same steps except for incubation with primary antibodies. Scale bars = 100 μm.

As shown in Figure 5, the effect of NT-4 on the cell numbers of the ICM and TE was investigated via differential immunostaining for SOX2 (ICM marker) and CDX2 (TE marker) in PA-derived blastocysts. In this experiment, total 21 blastocysts were used for each group. Each Hoechst-33342-positive cell (Total cell number of blastocysts) was classified as ICM or TE based on the predominant expression of SOX2 (Green) or CDX2 (Red), respectively. The number of CDX2-expressing cells (putative TE cells) was significantly higher (*p* < 0.05) in the NT-4-supplemented group (34.19 ± 1.9) than in the control group (28.24 ± 1.8). In contrast, both the number of SOX2-expressing cells (putative ICM cells) and the ICM/TE ratio were significantly lower (*p* < 0.01) in the NT-4-supplemented group (4.14 ± 0.4 and 13.97 ± 2.2, respectively) than in the control group (6.71 ± 0.6 and 26.53 ± 3.2, respectively) (Table 1).

**TABLE 1:** Effect of NT-4 supplementation during IVC on cell lineage specification of parthenogenetically-activated embryos.

	Group	
Parameter	Control	NT-4 treatment
Number of examined blastocysts	21	21
Average total cell number in blastocysts	42.29 ± 1.9	46.67 ± 1.8
Number of SOX2-expressing cells	6.71 ± 0.6 (16.39% ± 1.6%)	4.14 ± 0.4^**^ (9.12% ± 1.0%)
Number of CDX2-expressing cells	28.24 ± 1.8 (66.40% ± 2.5%)	34.19 ± 1.9^*^(73.77% ± 3.4%)
Number of cells without expression of SOX2 and CDX2	7.33 ± 1.0 (17.20% ± 2.0%)	8.33 ± 1.6 (17.12% ± 2.9%)
SOX2 to CDX2 ratio	26.53 ± 3.2	13.97 ± 2.2^*^

Data are given as mean ± SEM.

SOX2-expressing cells, cells with SOX2 expression which candidate to inner cell mass in pre-implantation embryos.

CDX2-expressing cells, cells with CDX2 expression which candidate to trophectoderm in pre-implantation embryos.

Asterisks indicate statistical significance (*p < 0.05 and **p < 0.01).

This experiment was replicated three times.

### 3.5 Effect of NT-4 supplementation during IVC on YAP1 expression of PA-derived blastocysts

**FIGURE 6:**
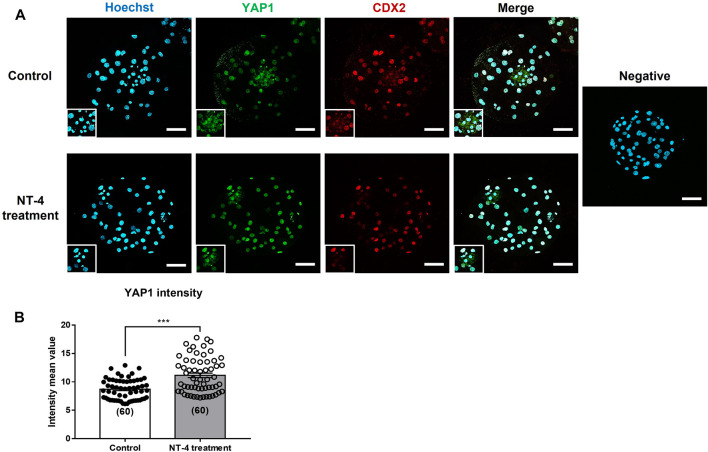
Effect of NT-4 supplementation during *in vitro* culture (IVC) on YAP1 localization of parthenogenetic activation (PA)-derived blastocysts. **(A)** NT-4 was added at 0 (Control) and 10 ng/mL during PA embryo development *in vitro*. Green (AF488) fluorescence labeling indicates YAP1. Red (AF594) fluorescence labeling indicates CDX2. Nuclei are counterstained with Hoechst-33342 (blue). A negative control followed all the same steps except for incubation with primary antibodies. Square markings indicate areas presumed to be ICM. Scale bars = 100 μm. **(B)** Quantification of YAP1 intensity in each PA-derived blastocyst group. Mean values of fluorescence intensities were quantified using the ZEN software program. Data were analyzed by Student’s t-tests. Number of blastocysts = 60. Asterisks indicate statistical significance (***p < 0.001)

As our results confirmed that NT-4 treatment during IVC promotes TE lineage specification rather than the ICM lineage in PA-derived blastocysts (Figure 3A; Figure 4A; Figure 5 and Table 1), we also investigated whether NT-4 supplementation during IVC affects YAP1 protein localization in the blastocysts using immunofluorescence (Figure 6A). Notably, the YAP1 protein was predominantly localized to the TE nuclei in porcine PA-derived blastocysts, and it was also present in the cytoplasm of ICM. The fluorescence intensity of YAP1 was significantly (*p* < 0.001) higher when porcine embryos were cultured with NT-4 compared to that of the control group (Figure 6B). Additionally, we investigated the inhibition of Hippo-YAP signaling during porcine IVC using 1 μM verteporfin (a YAP inhibitor) (Supplementary Figure S1). In the PA experiment, no significant (*p* > 0.05) difference in cleavage rates was observed between any of the groups. When the blastocyst formation pattern was confirmed, no significant (*p* > 0.05) difference was observed in the expanded blastocyst formation rate between the treatment and control groups. However, the expanded blastocyst formation rates of verteporfin (9.52% ± 3.9%) and NT-4 + verteporfin (17.6% ± 5.4%) groups were significantly (*p* < 0.05) lower than that of the NT-4-treated group (39.6% ± 7.9%). The total blastocyst formation rate of the PA embryos was significantly (*p* < 0.05) lower in the verteporfin-treated group (19.9% ± 6.2%) than in both the control (43.0% ± 4.4%) and NT-4-treated group (51.9% ± 7.7%). However, no significant (*p* > 0.05) difference was observed in the total blastocyst formation rate between the NT-4-treated and control groups.

## 4 Discussion

The purpose of this study was to investigate the effects of NT-4 supplementation during IVC of porcine PA embryos. NT-4 supplementation during IVC improved the developmental potential of porcine PA embryos by regulating genes related to apoptotic, TE lineages, and neurotrophin signaling. To the best of our knowledge, this study, for the first time, demonstrated that the presence of NT-4 in porcine PA-derived blastocysts and NT-4 supplementation during IVC contributed toward developing the TE lineage rather than the ICM during porcine early embryonic development.

Neurotrophins play an important role in the development of the reproductive system as well as the development of the central and peripheral nervous systems. During pregnancy, neurotrophins affect maternal implantation, placental angiogenesis, and fetal brain development (Sahay et al., 2017; D’Angelo et al., 2020). NGF, BDNF, NT-3, and NT-4 play essential roles during pregnancy in the placenta and fetus (Kanai-Azuma et al., 1997; Toti et al., 2006; Casciaro et al., 2009; Kawamura et al., 2009; Mayeur et al., 2010; Lim et al., 2017). In humans, placental NGF mRNA and protein are mainly localized to trophoblast cells, with NGF contributing toward successful implantation and placental angiogenesis (Toti et al., 2006). BDNF improves mouse blastocyst development and reduces apoptosis in blastocysts (Kawamura et al., 2007). Moreover, in mice, BDNF and NT-4 mRNA and protein are localized in TE cells but not in ICM cells (Kawamura et al., 2007). In pigs, BDNF induces the development of endometrial luminal epithelial cells for implantation and maintenance of pregnancy by interacting with TrkB to activate the PI3K and MAPK pathways and inhibit ER stress (Lim et al., 2017). In a bovine study, supplementation of the IVC medium with BDNF (40 ng/mL) improved embryonic development after PA and IVF (Yi et al., 2008), indicating that the BDNF protein was localized in bovine TE cells and promoted the proliferation of granulosa cells. Only one study on NT-3 expression in the placenta has confirmed it in human placenta during different trimesters, suggesting that NT-3 may play a role in placental and fetal development (Casciaro et al., 2009). However, unlike other neurotrophins, little is known about the role of NT-4 in the development of placenta and fetus during pregnancy.

Various autocrine, paracrine, and endocrine growth factors, such as the epidermal growth factor, insulin-like growth factor-I, and granulocyte-macrophage colony-stimulating factor, are commonly involved in mammalian pre-implantation embryonic development (Hardy and Spanos, 2002). Although neurotrophins are also growth factors that regulate the pre-implantation embryonic development and implantation in mice or humans, their effects on porcine pre-implantation embryonic development have not been studied. In the present study, we first identified the presence of NT-4 and its receptors in porcine PA-derived blastocysts. Receptor tyrosine kinases (RTKs), act as cell surface receptors that transmit external signals into cells through various signaling pathways, have been shown to localize in other compartments within cells, such as the nucleus and mitochondria, in addition to localizing on the cell surface (Huo et al., 2014). For example, one of the RTKs, TrkA, has been demonstrated to localize to both the nuclei and cytoplasm of bovine blastocysts (Munoz et al., 2009). Similar to the findings of Munoz et al. (Munoz et al., 2009), we also found that total-TrkB is localized to the nuclei within the blastocyst. In contrast to the localization of total-TrkB, phospho-TrkB was localized to the cell surface and cytoplasm of porcine PA-derived blastocysts. The localization of total-TrkB and p75^NTR^ in the nuclei of porcine blastocysts, as revealed by our study, is similar to that of previous studies; this is indicative of the ability of intact RTK to translocate into the nuclei in a ligand-dependent manner (Carpenter, 2003; Krolewski, 2005; Massie and Mills, 2006). Since the present study is the first to show the localization of neurotrophic factors and their receptors in porcine PA blastocysts, further studies are needed to confirm their localization patterns across different stages of porcine embryonic development. According to our previous study, NT-4, total-TrkB, phospho-TrkB, and p75^NTR^ were localized in both the nuclei and cytoplasm of porcine cumulus cells, whereas they were only localized to ooplasms of *in vitro* matured-porcine oocytes (Kim et al., 2022). Therefore, our present study demonstrated that the localization patterns of NT-4 and its receptors are cell-type specific. In contrast to the mouse and bovine studies (Kawamura et al., 2007; Yi et al., 2008), NT-4, total-TrkB, phospho-TrkB, and p75^NTR^ proteins were observed to be distributed in both ICM and TE in porcine PA-derived blastocysts. Therefore, we demonstrated that there was a difference between species in the expression of NT-4 protein in the blastocysts. To the best of our knowledge, this is the first study to confirm the identification of NT-4 and its receptor proteins in porcine blastocysts. This finding suggested that NT-4 has the potential to also affect porcine embryonic development.

In this study, we confirmed that the addition of NT-4 during IVC promoted the developmental competence of porcine PA-derived embryos. Among the various concentrations (0, 1, 10, and 100 ng/mL) of NT-4 supplementation, only 10 ng/mL NT-4 supplementation to the IVC medium significantly increased the rate of blastocyst formation. Therefore, the optimal concentration of NT-4 in porcine IVC was determined to be 10 ng/mL. Unlike IVF, in which a major factor is successful penetration of a single sperm into an oocyte, PA is the process by which a single oocyte develops into an embryo without any paternal contribution and meiotic chromosomal loss (Paffoni et al., 2008). IVF has many other intrinsic and extrinsic variables that affect the efficiency of embryonic development, and the high incidence of polyspermy remains a major obstacle, especially with respect to porcine IVF (Wang et al., 1991). Therefore, we investigated the effect of NT-4 on porcine parthenogenetic embryonic development without the exogenous influence of sperm. However, since parthenogenesis does not naturally occur in mammals, it is necessary to investigate the effect of NT-4 during IVC on the development of porcine IVF- or somatic cell nuclear transfer (SCNT)-derived embryos. In particular, it would be very interesting to compare and analyze differences in embryonic development from different species and origins. Previous studies have investigated chromatin (Deshmukh et al., 2012) or transcriptome (Van Der Weijden et al., 2021) dynamics in porcine embryos produced *in vivo* and *in vitro*. A recent study has shown that lipid metabolism varies by origins (IVF and PA) during bovine embryonic development (Lipinska et al., 2023). Their study (Lipinska et al., 2023) has shown that lipid parameters of IVF and PA bovine embryos differ from each other at the most critical stages of embryonic development (zygotes, 8-16 cells, and blastocysts), indicating that PA embryos may have dysregulation of lipid metabolism (higher lipid accumulation) during embryonic development. Therefore, a comparative analysis of differences in porcine embryonic development from different origins (*in vivo*, PA, IVF, and SCNT) should be considered in future studies.

To investigate the effect of NT-4 on transcription patterns of blastocysts during IVC, qRT-PCR was performed by sampling PA-derived blastocysts. We confirmed that NT-4 reduced apoptosis in the porcine blastocysts as both the pro-apoptotic *BAX* gene and the *BAX*/*BCL2L1* ratio were downregulated, and the anti-apoptotic *BCL2L1* gene was upregulated. Similar to our study, Kawamura et al. (Kawamura et al., 2007) demonstrated that BDNF (10 ng/mL), which is known to have physiologically similar functions to NT-4, enhanced pre-implantation embryonic development and reduced blastocyst apoptosis in mice. Although no significant difference was observed in the mRNA expression of ICM-related genes (*POU5F1* and *SOX2*) in blastocysts during NT-4 treatment in porcine IVC, the mRNA transcript levels of *CDX2*, *PPAG3*, and *GATA3* among the TE-related genes were significantly increased in the NT-4-supplemented group. Caudal-related homeobox protein 2 (CDX2) is one of the transcription factors that exhibits differential expression inside and outside the blastomeres, and it is well-known that *Cdx2* expression before blastocyst formation in mice plays a pivotal role in TE specification (Strumpf et al., 2005; Jedrusik et al., 2008). Generally, GATA binding protein 3 (GATA3) activates CDX2 and is essential for TE lineage development (Home et al., 2009). In pigs, CDX2 is first observed in some TE cells immediately after cavitation in day-5 embryos and later remains only in TE cells as embryonic development continues (Kuijk et al., 2008; Bou et al., 2017). In bovine and porcine embryos, *CDX2* and *GATA3* mRNA transcripts are distributed in all the cells of blastocysts regardless of ICM and TE; however, these are known to be mainly distributed in TE cells after the transition from the blastocyst stage to the pre-implantation embryonic elongation stage (Fujii et al., 2010; Fujii et al., 2013). Therefore, these two transcription factors are used as porcine TE-specific markers. Porcine pregnancy-associated glycoprotein 3 (PPAG3) is a glycoprotein expressed in the placenta, and it has also been used as a trophoblast marker in pigs (Arcuri et al., 2021). Combined with the findings of our study, these demonstrate that NT-4 supplementation during IVC upregulates the mRNA expression of some TE-related genes (*CDX2*, *PPAG3*, and *GATA3*) in PA embryos, suggesting that NT-4 contributes more to TE lineage development than to ICM lineage development in porcine early embryonic development.

We also confirmed that NT-4 supplementation in the IVC medium regulated the mRNA expression of neurotrophin signaling-related genes (i.e., *NGFR*, *NFKB1*, *FURIN*, *ERK1*, *ERK2*, and *Akt1*) in PA-derived blastocysts. Among these neurotrophin-related genes, *NFKB*, *FURIN*, and *ERK1* transcripts were significantly upregulated (*p* < 0.05) in NT-4-supplemented blastocysts. *NFKB1* promotes cell survival as a downstream effector of the neurotrophin pathway (Karin and Lin, 2002; Guo et al., 2021). NT-4 supplementation during IVC significantly increases the level of the *NFKB1* transcript, a downstream effector of the *NGFR* gene during pre-implantation embryonic development. This implies that the upregulation of the *NFKB1* transcript through neurotrophin/p75^NTR^ signaling improves the developmental capacity of pig embryos. Furin is an essential mammalian proprotein convertase that catalyzes the proteolytic maturation of various proproteins, including converting proneurotrophins to mature neurotrophins in the secretory pathway (Zhang et al., 2022). It is highly expressed in human and monkey invasive extravillous cytotrophoblasts; additionally, it is reportedly essential for the invasion of trophoblasts into the maternal endometrium (Zhou et al., 2009). Furin is also an important conversion enzyme that promotes the functioning of the insulin-like growth factor-1 (Duguay et al., 1995) and vascular endothelial growth factor (Siegfried et al., 2003) associated with TE fusion and trophoblast syncytialization. Hence, supplementation with NT-4 during porcine IVC has the potential to promote trophoblast differentiation by increasing *FURIN* mRNA levels in porcine embryos. BDNF binds to TrkB, inducing signaling cascades involving MAPK/ERK and phosphoinositide 3-kinase (PI3K)/Akt pathways to promote neuronal survival (Kaplan and Miller, 2000; Patapoutian and Reichardt, 2001). In mice, BDNF promotes pre-implantation embryonic development by mediating the PI3K/Akt pathway rather than the MAPK/ERK pathway (Kawamura et al., 2007). It also affects placental development by promoting trophoblast growth and survival via the TrkB/ERK pathway (Kawamura et al., 2009). BDNF also enhances bovine granulosa cell proliferation through the TrkB/Akt and MAPK/ERK pathways; it also increases progesterone synthesis through the TrkB/Akt pathway (Chen et al., 2019). In pigs, BDNF activates TrkB and mediates PI3K/Akt and MAPK (ERK1/2, JNK, and P38) signaling pathways to promote endometrial luminal epithelial cell proliferation through the upregulation of *PCNA* and *CCND1* (Lim et al., 2017). However, so far, the effects of NT-4 on mammalian embryonic development and proliferation of trophoblast or endometrial cells have not been studied. Therefore, it is necessary to investigate NT-4-related signaling pathways that mediate and affect pre-implantation embryonic development in pigs using specific PI3K or MEK inhibitors.

Mammalian embryogenesis is characterized by self-organized spatial patterning, which determines the fate of the cells of different lineages. Pre-implantation embryos develop from the zygote to the blastocyst, which comprises the ICM and TE cells that become the fetus and placenta, respectively (Reik et al., 2003). The first lineage segregation in pigs begins on embryonic day 5, specifically, the late morula stage (Zhi et al., 2022). SOX2 is a genuine porcine ICM marker that plays an essential role in the first lineage specification through an asymmetric distribution before the morula stage during porcine pre-implantation embryonic development (Lee et al., 2021). In mice, OCT4 (Palmieri et al., 1994; Nichols et al., 1998; Frum et al., 2013) and CDX2 (Strumpf et al., 2005) are used as ICM and TE markers, respectively. However, in pigs, OCT4 is co-expressed with CDX2 in blastocysts and is present in both ICM and TE cells (Kuijk et al., 2008). Overall, previous studies have demonstrated that SOX2 and CDX2 can be used as ICM and TE markers in porcine blastocysts, respectively (Liu et al., 2015; Yoon et al., 2019b). In this study, we immunostained and analyzed blastocysts with SOX2 and CDX2 to determine the effect of NT-4 on the first lineage specification in porcine early embryonic development. As a result of differential immunostaining, cells expressing neither SOX2 nor CDX2 were observed in the blastocysts; these cells were presumed not to belong to either the epiblast or TE. An example of such cells is hypoblasts, which constitute another part of the ICM (Yoon et al., 2019b). However, this finding needs to be further confirmed through the detection of GATA4 and GATA6, which are markers for the presence of the hypoblasts in pigs (Zhi et al., 2022). Interestingly, NT-4 supplementation during IVC significantly (*p* < 0.05) increased the number of CDX2-expressing cells in the blastocysts while significantly (*p* < 0.01) decreasing the number of SOX2-expressing cells and the ICM/TE ratio (SOX2/CDX2 ratio). Placentation begins with the formation of TE cells from pre-implantation blastocysts. These TE cells differentiate into trophoblast cells after implantation (Li et al., 2019). As the dysfunction of trophoblasts can lead to adverse pregnancy outcomes including pre-eclampsia and pre-term birth (Sahay et al., 2017), placental development is important, which requires the stimulation of differentiation into the TE cells during pre-implantation embryonic development. During pregnancy, neurotrophins (NGF and BDNF) play a very important role in the successful implantation of the embryo and placental and fetal development (Toti et al., 2006; Nico et al., 2008; Mayeur et al., 2010; Zheng and Shao, 2012; Dhobale, 2017). Therefore, we suggest that NT-4 supplementation can regulate the gene expression of *CDX2* and may promote the TE lineage specification during porcine early embryonic development. However, further studies are needed to clarify whether NT-4 also affects placental growth and development.

Upon fertilization, mammalian zygotes undergo symmetric and asymmetric cell division, and the first embryonic lineage is established through morula compaction and cavitation (Guo et al., 2010). We further confirmed that the relative mRNA transcript levels regulating the Hippo and Notch signaling pathways are related to the first-cell fate decision of embryos. We confirmed that the *YAP1* transcript level was significantly increased (*p* < 0.05), whereas *LATS2* transcript level was significantly decreased (*p* < 0.01) in NT-4-supplemented blastocysts than in the control. YAP localization depends on the temporal induction of large tumor suppressor (LATS) kinase activity, and it regulates embryonic lineage specification (Lorthongpanich et al., 2013; Wicklow et al., 2014). In mice, when Hippo signaling is activated in the inner cells (ICM lineage) of pre-implantation embryos, LATS1/2-dependent YAP phosphorylation occurs in the cytoplasm, thereby preventing the accumulation of YAP in the nucleus. Therefore, *Cdx2* is repressed and *Sox2* is expressed in the ICM cells (Sasaki, 2017). In contrast, when Hippo signaling is inactivated in the outer cells (TE lineage) of pre-implantation embryos, LATS1/2 kinase activity is reduced, and unphosphorylated YAP accumulates in the nucleus. YAP then interacts with the TEA domain transcription factor 4 (TEAD4), and these active TEAD4-YAP complexes upregulate *Cdx2* transcription in the TE cells. Although our results did not show a significant difference in the *TEAD4* transcript levels in blastocysts when NT-4 was treated during IVC, it appears that the *CDX2* transcript level was significantly increased in blastocysts while *YAP1* was significantly upregulated and *LATS2* was downregulated. However, when NT-4 was added to the IVC medium, no significant difference was observed in the Notch effector *HES1* mRNA transcript level. Previous studies have shown that the Notch signaling pathway induces CDX2 expression in the morula stage of mouse embryos, but this process has not been confirmed in other species (Rayon et al., 2014; Watanabe et al., 2017; Menchero et al., 2019). Our results suggest that NT-4 may induce the CDX2 expression by interacting with the Hippo-YAP signaling pathway rather than the Notch signaling pathway, and regulate early lineage specification in porcine embryos. Our findings also help explain why the mechanisms regulating lineage specification in mammalian embryos differ among species. Similar to the qPCR data, the immunostaining results demonstrated that the addition of NT-4 during IVC significantly (*p* < 0.001) increased YAP1 expression in blastocysts. Therefore, the addition of NT-4 during porcine IVC enhanced early embryonic developmental competence while increasing the expression of YAP1 in the embryos; NT-4 was also confirmed to be involved in early lineage segregation.

We also evaluated the inhibitory effect of Hippo-YAP signaling and the developmental potential of PA embryos using verteporfin during IVC. Maternal YAP in porcine embryos is required for normal blastocyst development (Cao et al., 2019); additionally, the inhibition of the YAP/TEAD interaction using 1 μM verteporfin for 7 days (168 h) of IVC significantly decreased the developmental capacity of porcine embryos that reached the 8-cell and blastocyst stages. In this study, when using the YAP inhibitor during porcine IVC, no significant (*p* > 0.05) differences were observed throughout the cleavage stage including the 8-cell stage, but the rate of blastocyst formation, similar to the findings of a previous study (Cao et al., 2019), was significantly (*p* < 0.05) decreased compared to that of the control group. However, no significant (*p* > 0.05) difference was observed in the developmental competence of PA embryos between the control and NT-4-treated groups. In a bovine study, 0.5% DMSO supplementation during IVM increased first polar body extrusion rate and improved subsequent embryonic development after IVF (Ynsaurralde-Rivolta et al., 2020). In pigs, short- (20 h) or long-term (144 h) exposure to DMSO, a vehicle at low concentrations (0.1%), was not toxic to the development of *in vitro*-produced IVF embryos (Lucas et al., 2021). As verteporfin and NT-4 were dissolved in DMSO and DPBS, respectively, we dissolved both DMSO (0.1%) and DPBS (2%) in the PZM-3 medium as vehicles for the YAP inhibition experiment. However, no significant difference was observed in developmental capacity between the NT-4 treated group and the control groups treated with both DPBS and DMSO as the vehicles (Supplementary Figure S1), unlike that in the PA experiment in which only DPBS was used as the vehicle (Figure 2). Thus, this study suggests that DMSO has some effect on the action of NT-4 protein during IVC; however, the specific mechanisms underlying this effect need to be elucidated. From these results, it is clear that the Hippo-YAP signaling plays a very important role in the normal development of porcine embryos and that NT-4 does not compensate for the inhibitory effect of YAP but can promote the expression of YAP1 while contributing to TE lineage specification during porcine PA embryonic development.

In conclusion, to the best of our knowledge, this is the first study to confirm the effect of NT-4 on mammalian embryonic development. NT-4, TrkB, and p75^NTR^ proteins were localized in both ICM and TE in porcine PA-derived blastocysts. The optimal concentration of NT-4 to improve the porcine PA embryonic developmental competence was 10 ng/mL. NT-4 supplementation significantly increased in CDX2-expressing cells (putative TE cells) by inducing the transcription of the TE lineage markers (*CDX2*, *PPAG3*, and *GATA3* transcripts). NT-4 reduced blastocyst apoptosis by regulating the transcription of apoptosis-related genes and improved the blastocyst quality by interacting with the Hippo-YAP and MAPK/ERK pathways. NT-4 supplementation during IVC significantly increased and decreased the number of CDX2-expressing and SOX2-expressing cells, respectively, in the blastocysts and significantly decreased the ICM/TE ratio (SOX2/CDX2 ratio). NT-4 contributes toward promoting differentiation into the TE lineage rather than into the ICM lineage during porcine early embryonic development. In summary, 10 ng/mL NT-4 supplementation enhanced PA embryonic developmental competence by regulating the apoptosis-, TE lineage specification-, and neurotrophin signaling-related genes during *in vitro* porcine embryo development.

These findings provide novel and valuable insights into the molecular mechanisms of NT-4 that reduce apoptosis, improve blastocyst quality, and promote differentiation into TE cells during porcine embryo production *in vitro*. This study will aid in understanding the process of first cell lineage segregation in the porcine embryos. Whether the effect of NT-4 on ICM/TE lineage specification mimics the natural conditions of embryonic development *in utero* and *in vivo*, or whether it has any (i.e., beneficial or detrimental) effects on the embryo requires further investigation. The involvement of NT-4 in porcine early embryonic development, demonstrated in this study, may provide novel and interesting clues to the functional study of NT-4, which is relatively less studied than other neurotrophic factors.

Although this study investigated the role of NT-4 in porcine embryonic development after PA, the manner in which various neurotrophic factors other than NT-4 affect porcine early embryonic development remains unknown. In particular, it is necessary to confirm the combined effect of BDNF and NT-4 on early embryogenesis in pigs. Additionally, the importance of NT-4 during porcine early embryonic and placental development and its interactions with other environmental factors including neurotrophins require further investigation.

## Data Availability

The original contributions presented in the study are included in the article/Supplementary Material, further inquiries can be directed to the corresponding author.

## Supplementary material

Supplementary Figure S1 Effect of NT-4 and Hippo signaling during porcine *in vitro* culture (IVC). **(A)** Effect of NT-4 and Hippo signaling during IVC on embryonic development after parthenogenetic activation (PA). **(B)** Effect of NT-4 and Hippo signaling during IVC on the cleavage pattern of PA embryos at day 2. **(C)** Effect of NT-4 and Hippo signaling during IVC on the percentage of PA embryos that developed to the blastocyst stage at day 7. **(D)** Representative morphologies of porcine blastocysts from each group 7 days after PA. Scale bar = 100 µm. For all graphs, the value represents the mean ± SEM. Within each end point, bars with different letters (a, b, and c) are significantly (*p* < 0.05) different. Statistical significance was determined by one-way ANOVA. 1+C, 1 cell + fragmentation embryos; 2C, 2 cell; 4C, 4 cell; 8C, 8 cell; CL, cleavage; BL, blastocyst. The experiment was replicated four times.
