# Supplementary material for: Neurotrophin-4 promotes the specification of trophectoderm lineage after parthenogenetic activation and enhances porcine early embryonic development
Source: Front Cell Dev Biol. 2023 Jul 13;11:1194596. doi: 10.3389/fcell.2023.1194596 (PMC10373506; doi:10.3389/fcell.2023.1194596)
Supplement: Supplementary file 1 [file Table1.docx]

**Supplementary table 1.** Primer lists for qRT-PCR.

| **mRNA** | **Primer sequences** | **Product size**  **(bp)** | **GenBank**  **accession number** |
| --- | --- | --- | --- |
| ***POU5f1*** | F: 5’-CTCACTTTGGGGGTTCTCTT-3’ | 169 | NM_001113060 |
|  | R: 5’-TCTCTGCCTTGCATATCTCC-3’ |  |  |
| ***SOX2*** | F: 5’-AACAGCCCAGACCGAGTTAAG-3’ | 254 | NM_001123197 |
|  | R: 5’-ATGAGCGTCTTGGTTTTCCG-3’ |  |  |
| ***CDX2*** | F: 5’-CTGTTTGGGTTGTTGGTCTG-3’ | 95 | NM_001278769 |
|  | R: 5’-CCCACTCCCTTCACCATATC-3’ |  |  |
| ***TEAD4*** | F: 5’-GATCAGGCAGCTAAGGACAA-3’ | 87 | NM_001142666.1 |
|  | R: 5’-TTTATTGTGGAAGGCTGAGG-3’ |  |  |
| ***PPAG3*** | F: 5'-AGTACCAGGTCCGATGTTG-3' | 224 | NM_213809 |
|  | R: 5'-TCCTCGATCAAAAACAGTGA-3' |  |  |
| ***BAX*** | F: 5’-TGCCTCAGGATGCATCTACC-3’ | 199 | XM_013998624 |
|  | R: 5’-AAGTAGAAAAGCGCGACCAC-3’ |  |  |
| ***BCL2L1*** | F: 5’-AATGACCACCTAGAGCCTTG-3’ | 182 | NM_214285 |
|  | R: 5’-GGTCATTTCCGACTGAAGAG-3’ |  |  |
| ***YAP1*** | F: 5’-CAATGACGACCAATAGCTCA-3’ | 185 | XM_021062706 |
|  | R: 5’-CTGTGAAGGTAGGGTGCTTT-3’ |  |  |
| ***LATS2*** | F: 5’-GTGGGTGGTGAAGCTCTACT-3’ | 177 | XM_021065129 |
|  | R: 5’-ATCCTGTGGACACTCTCGAT-3’ |  |  |
| ***GATA3*** | F: 5’-GGACAGAATCGACCCCTTAT-3’ | 197 | XM_021064073 |
|  | R: 5’-CCTTCCTTCTTCATGGTCAG-3’ |  |  |
| ***TFAP2C*** | F: 5’-GTCGCTCCTCAGCTCTACAT-3’ | 246 | NM_001123201 |
|  | R: 5’-AAGTGAACAGCCTCACCTTC-3’ |  |  |
| ***DAB2*** | F: 5’-AACAGGCAGAGCCATTAGTC-3’ | 181 | XM_021076652 |
|  | R: 5’-ATCTGGTCAACACCCAGTTT-3’ |  |  |
| ***HES1*** | F: 5’-GAAAGTCATCAAAGCCCATC-3’ | 160 | NM_001195231 |
|  | R: 5’-AGGTGCTCCACTGTCATTTC-3’ |  |  |
| ***ERK1*** | F: 5'-ATCACAGTGGAGGAAGCACT-3' | 202 | XM_021088019 |
|  | R: 5'-GAGGCATCTGTCCAGGTTAG-3' |  |  |
| ***ERK2*** | F: 5'-AGTCCATCGACATCTGGTCT-3' | 240 | XM_021088019 |
|  | R: 5'-GAGCTTTGGAGTCAGCATTT-3' |  |  |
| ***Akt1*** | F: 5'-CTACAACCAGGACCACGAGA-3' | 208 | NM_001159776 |
|  | R: 5'-CTCATACACATCCTGCCACA-3' |  |  |
| ***CTGF*** | F: 5’-AACTATGATGCGAGCCAACT-3’ | 174 | NM_213833 |
|  | R: 5’-ATGTTCTCTTCCAGGTCAGC-3’ |  |  |
| ***NGFR*** | F:5'-TGGAGATGGAGATGATATGGA-3' | 316 | NM_001244828 |
|  | R: 5'-GGCAATCTCCAATTAGAAGC-3' |  |  |
| ***NFKB1*** | F: 5'-CTACCAGACACCCTTGCACT-3' | 222 | NM_001048232 |
|  | R: 5'-ATAGCGTTCAGACCTTCACC-3' |  |  |
| ***FURIN*** | F: 5’-GCTCACCCTGTCCTACAATC-3’ | 236 | XM_021098919 |
|  | R: 5’-CGTCCCATAGAACAAAACCT-3’ |  |  |
| ***RN18S*** | F: 5’-CGCGGTTCTATTTTGTTGGT-3’ | 219 | NR_046261 |
|  | R: 5’-GGTCATTTCCGACTGAAGAG-3’ |  |  |

**Supplementary table 2.** Antibody lists for immunofluorescence staining.

| **Antibody** | **Host** | **Dilution** | **Cat.** |
| --- | --- | --- | --- |
| **Anti-NT-4** | Mouse | 1:100 | NBP1-47897 |
| **Anti-TrkB** | Mouse | 1:100 | sc-377218 |
| **Anti-Phospho TrkB** | Rabbit | 1:100 | PA5-36695 |
| **Anti-p75^NTR^** | Mouse | 1:100 | 14-9400-82 |
| **Anti-SOX2** | Mouse | 1:100 | #4900 |
| **Anti-CDX2** | Rabbit | 1:100 | #12306 |
| **Anti-YAP1** | Mouse | 1:100 | H00010413-M01 |
| **Alexa Fluor 488** | Mouse | 1:200 | A11029 |
| **Alexa Fluor 594** | Rabbit | 1:200 | A21207 |
