# Supplementary figures and images for: Neurotrophin-4 promotes the specification of trophectoderm lineage after parthenogenetic activation and enhances porcine early embryonic development
Source: Front Cell Dev Biol. 2023 Jul 13;11:1194596. doi: 10.3389/fcell.2023.1194596 (PMC10373506; doi:10.3389/fcell.2023.1194596)

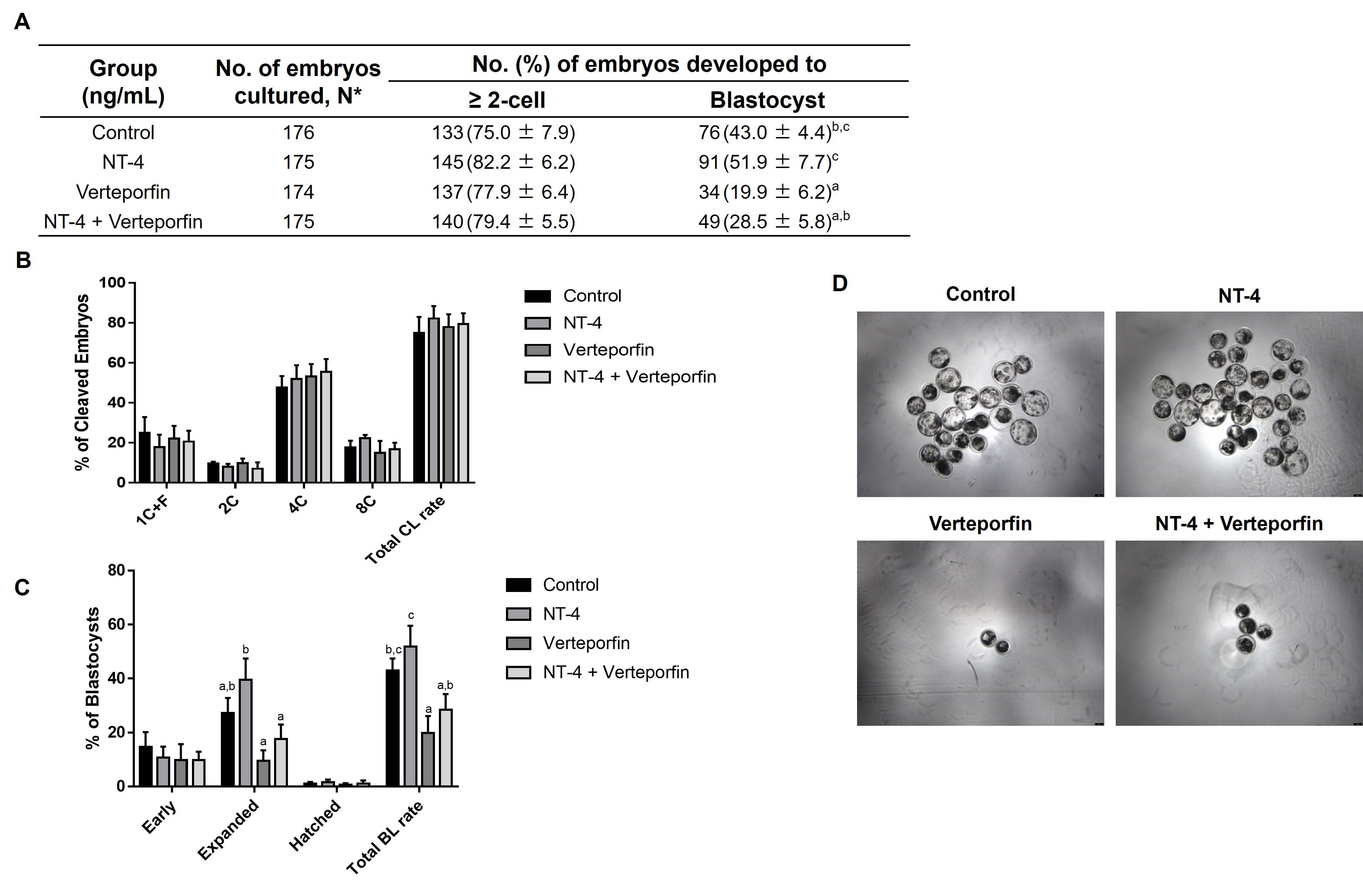

Supplement: Supplementary file 2 [file Image1.TIF]
